# Supplementary material for: Methylphenidate Promotes Premature Growth Plate Closure: In Vitro Evidence
Source: Int J Mol Sci. 2023 Feb 20;24(4):4175. doi: 10.3390/ijms24044175 (PMC9968202; doi:10.3390/ijms24044175)
Supplement: Supplementary file 1 [file ijms-24-04175-s001.zip › ijms-2111204-supplementary.pdf]

Supplementary Figure S1

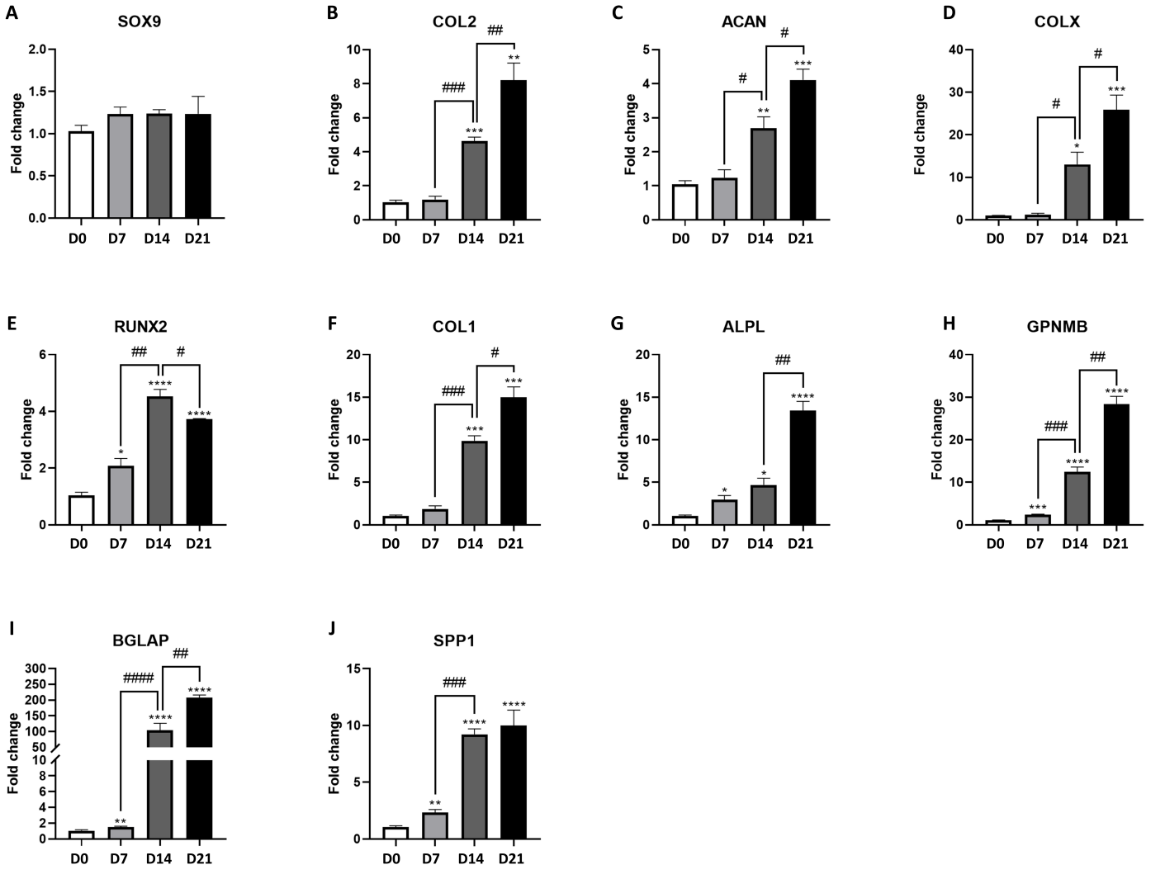

Figure S1. Relative expression levels of chondrocyte (a-d) and osteoblast (e-j) markers in ATDC5 cells throughout the 21 days of differentiation only in the control condicion. This is to prove that the differentiation was performed correctly. SOX9: SRY-box transcription factor 9; COL2: type II collagen; ACAN: aggrecan; RUNX2: Runt-related transcription factor 2; COL1: type I collagen; ALPL: alkaline phosphatase; GPNMB: osteoactivin; BGLAP: osteocalcin; SPP1: osteopontin. Bars represent means with SEM (n = 3). *: statistically different vs. day 0 ($p < 0.05$); **: $p < 0.01$; ***: $p < 0.001$; ****: $p < 0.0001$; #: statistically different vs. untreated control from the same day ($p < 0.05$); ##: $p < 0.01$; ###: $p < 0.001$; ####: $p < 0.0001$.

Supplementary Figure S2

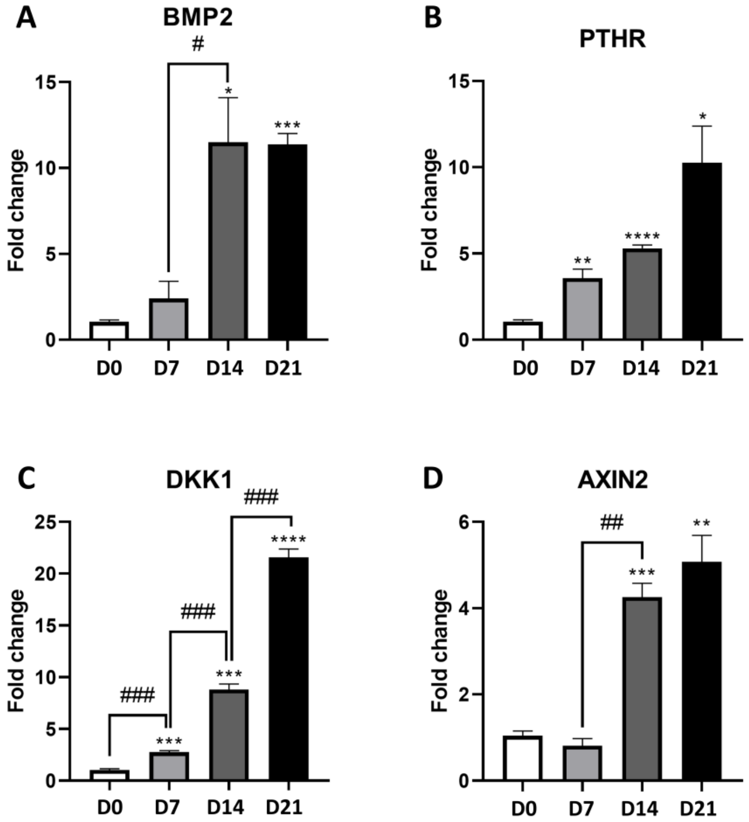

Figure S2. Relative expression levels of BMP2 (a), PTHR (b) and Wnt signalling-related genes (c-d) in ATDC5 cells throughout the 21 days of differentiation only in the control condition. This is to prove that the differentiation was performed correctly. BMP: Bone Morphogenetic Protein 2; PTHR: Parathyroid Hormone 1 Receptor; DKK1: Dickkopf WNT Signaling Pathway Inhibitor; AXIN2: Axis Inhibition Protein 2. Bars represent means with SEM (n = 3). *: statistically different vs. day 0 ($p < 0.05$); **: $p < 0.01$; ***: $p < 0.001$; ****: $p < 0.0001$; #: statistically different vs. untreated control from the same day ($p < 0.05$); ##: $p < 0.01$; ###: $p < 0.001$.
